# Analysis of Palliative Care Utilization and Medical Expenses among Patients with Chronic Diseases in Taiwan: A Population-Based Cohort Study

**DOI:** 10.3390/ijerph191912646

**Published:** 2022-10-03

**Authors:** Hui-Mei Lin, Yen-Chun Huang, Chieh-Wen Ho, Mingchih Chen

**Affiliations:** 1 Taipei City Hospital, RenAi Branch Nursing Supervisor, Taipei 106, Taiwan; 2 Graduate Institute of Business Administration, Fu Jen Catholic University, New Taipei City 242, Taiwan; 3 Artificial Intelligence Development Center, Fu Jen Catholic University, New Taipei City 242, Taiwan; 4 Department of Life Science, National Taiwan University, Taipei City 116, Taiwan

**Keywords:** chronic disease, chronic obstructive pulmonary disease, hypertension, palliative care, tranquility, World Health Organization

## Abstract

Palliative care (PC) is an important alternative treatment for patients with chronic diseases, particularly for those in the later stages of disease progression. This is because these diseases are often irreversible, with progressive worsening of symptoms. By encouraging the use of tranquility resources for good death and spiritual relief, PC can reduce the physical and psychological burden on patients at the end of their lives. Currently, most discussions on PC have focused on patients with cancers, and few have further discussed the differences in medical expenses between PC and emergency treatment in patients with chronic diseases at the end of their lives. This study analyzed the top three chronic diseases in patients who used PC resources in the past decade and identified the impact of emergency treatment on mean survival time and medical expenses based on the medical records from the National Health Insurance Research Database. In total, 4061 patients with chronic diseases who were admitted to hospice wards were included in this study; of them, 85 patients still received emergency treatment, including urinary catheterization, nasogastric intubation, and respirator use, at the end of their lives. The mean survival time of patients aged 50–64 years who received emergency treatment was longer than that of the same age group who did not receive emergency treatment. Different comparisons of the mean survival time and medical expenses using real-world data provides important insights regarding PC management that may assist in establishing health policies in the future.

## 1. Introduction

Chronic diseases, such as diabetes, hypertension, and asthma, are defined according to the biomedical disease classification. The World Health Organization (WHO) states that chronic diseases are not passed from person to person, but they are conditions that last for a long period and generally show slow progression. Chronic diseases are often irreversible, can progressively worsen symptoms, and can gradually limit the patients’ quality of life and ability to function [1]. As the population gradually grows older, chronic diseases are expected to become one of the most common health issues. Additionally, one chronic disease is usually combined with multiple chronic diseases as the patient ages [2,3]. Hence, >75% of the population in middle- and high-income countries are likely to die from one or multiple chronic diseases [1]. Chronic diseases account for more than half of all deaths and for approximately 70% of the total medical expenditure in Australia [2]. Among the top 10 causes of death named by the Taiwan Ministry of Health and Welfare in 2017, 56% were chronic diseases [4].

Among several chronic diseases, hypertension is the most significant risk factor for premature cardiovascular diseases and mortality [1]. Moreover, chronic obstructive pulmonary disease (COPD) is the third leading cause of mortality and the fifth leading cause of disability worldwide, with an estimated prevalence of 9–10% in adults aged 40 years [5,6,7,8]. The National Health Council of the United States reported that a cumulative annual economic burden of USD 1.3 trillion borne by the United States was due to the following seven most prevalent chronic conditions: cancer, diabetes, hypertension, stroke, heart disease, pulmonary conditions, and mental illness [9]. With the increase in their prevalence in the world’s population, chronic diseases have become the most challenging issues for public health worldwide [8,9,10].

Illness progression and comorbidity development would increase medical care requirements and death possibilities when chronic diseases are poorly controlled [11]. Chronic diseases have gradually become a healthcare economic burden worldwide [12,13]. In addition, treatments vary for different chronic diseases, leading to higher medical expenditures. Patients usually suffer from chronic diseases for a long time before their death, which causes mental and physical indisposition in these patients and their family members. Advanced medical techniques extend patients’ lives; however, they only prolong suffering for some patients with terminal illnesses. According to the characteristics of chronic diseases, disease development refers to the gradual and irreversible worsening of the patient’s health condition. In the advanced stages of chronic diseases, palliative care (PC) can be considered an alternative treatment in addition to continuous active therapy. It can not only offer patients with good death but also reduce their suffering caused by excessive life-sustaining or invasive interventions in patients who are terminally ill [14]. According to the WHO definition, PC is an approach that improves the quality of life of patients and their family members who face life-threatening illnesses by preventing and reducing hardship through the early identification and flawless estimation and treatment of pain and other physical, psychological, and spiritual issues [15]. The American Society of Clinical Oncology suggested that these dying patients should not receive active treatments. Life-prolongation therapy not only increases medical costs but also wastes medical resources when death is inevitable [16].

The main concept of PC involves improving the quality of life and alleviating the physical, psychological, and spiritual suffering of patients with life-threatening illnesses; this concept has been gradually assented and accepted by the public [17,18,19]. A previous study reported that PC is appropriate for treating patients with terminal cancer in Taiwan [20]; however, it should be provided not only to patients with severe cancer in the life-limiting stage but also to those with chronic health problems, mental illnesses, dyspnea, depression, anxiety, and multisymptomatic problems. The purpose of PC fits every patient with severe illness in the final stage. Patients with potentially curable diseases should receive dedicated PC services along with active treatment. The values of PC include emphasizing the dignity of life, improving quality of life, lowering costs of treatment, and reducing average duration and expenditure of hospitalization [21]. In Asia, PC was first promoted in Taiwan in 2008. Later, PC memorandum and its willing mark on the health identification card were established. The Taiwan National Health Insurance Administration 2012 provided family PC consultation fees to encourage family consultations, allowing inpatients to effectively access PC services through conference among the medical team, patient, and family members. This provides an opportunity to receive PC at their end of life, thereby enhancing the quality of good death in these patients [22,23,24,25,26]. The WHO suggested that PC services should be activated while lengthening life to provide a holistic perspective [27].

The main purposes of PC include satisfying the needs of patients and their family members to ease their pain, improving the patients’ dignity at the end of life, and reducing unnecessary treatments [28]. In PC consultations, the patient, family members, and medical team often discuss hospice care plans suitable for the patient. Moreover, the paramedical staff can help reduce the mental burden on the patient and assist grieving family members after the patient’s death [12,18,29,30].

In Asian countries, Confucianism and filial piety have emphasized the relationship between family members and patients. Thus, family members usually actively discuss with the physician regarding important decisions to reduce mental stress and burden on the patient; however, they often tend to ignore the feelings and physical suffering of the patient [23,25]. A previous study reported that inappropriate end-of-life care may cause the following problems: unnecessary hospitalizations, poor symptom management, patient death at unpreferable places, and lack of an opportunity to discuss the issue of death with their families [2].

Most countries have emphasized the careful observation of patients with terminal cancer. Meanwhile, only few studies have investigated the chronic diseases of patients [2,21]. Chronic diseases require long-term observation as they cause a decline in body functions if patients have a poor quality of life and increased number of acute care episodes, which are unpredictable [2,31]. However, patients with chronic diseases do not receive adequate PC, thus reducing the quality of their final life, months, weeks, or days [5,7]. Hence, hospitals must establish systematic disease management and provide appropriate PC for dying patients with chronic diseases [2].

The present study aimed to analyze the PC utilization of patients with the three most common chronic diseases, namely hypertension, hyperlipidemia, and COPD, as well as the resulting medical expenses based on the medical records from the National Health Insurance Research Database (NHIRD). The NHIRD is one of the most reliable and comprehensive databases worldwide, and approximately 98% of Taiwanese individuals participate in the National Health Insurance (NHI) program. This study included data of dead patients with chronic diseases who received PC between 2009 and 2019 from the NHIRD. The study results can be regarded as a reference to encourage terminal patients to utilize PC services and to promote decision makers to include chronic diseases in Chronic Care Programs. Meanwhile, as the population gradually grows older, an increased number of dying patients with chronic diseases using PC is expected. We believe that this study can offer some effective advice to these terminally ill patients for improving their quality of life while reducing the medical expenditure of unnecessary treatments in the life-limiting stage.

## 2. Materials and Methods

### 2.1. Data Source

The NHI program was established in 1995, covering approximately 99% of the population in Taiwan. The NHIRD contained personal medical and extensive healthcare information of 23 million individuals, including primary demographic characteristics; original clinical record; inpatient time; surgery; treatment and medication; expenditure; and diagnostic code, based on the International Classification of Disease, 9th and 10th Revisions, Clinical Modification (ICD-9-CM/ICD-10-CM); the 10th Revision has been initiated since 2016. The NHIRD provides the details of a comprehensive and long-term follow-up period of the NHI program for each beneficiary. All personal information was anonymized and deidentified before access. The research was approved by the Ethics Institutional Review Board of Fu Jen University in Taiwan (IRB number: C108121), and the requirement for obtaining patient informed consent was waived.

### 2.2. Study Population

**Figure 1:**
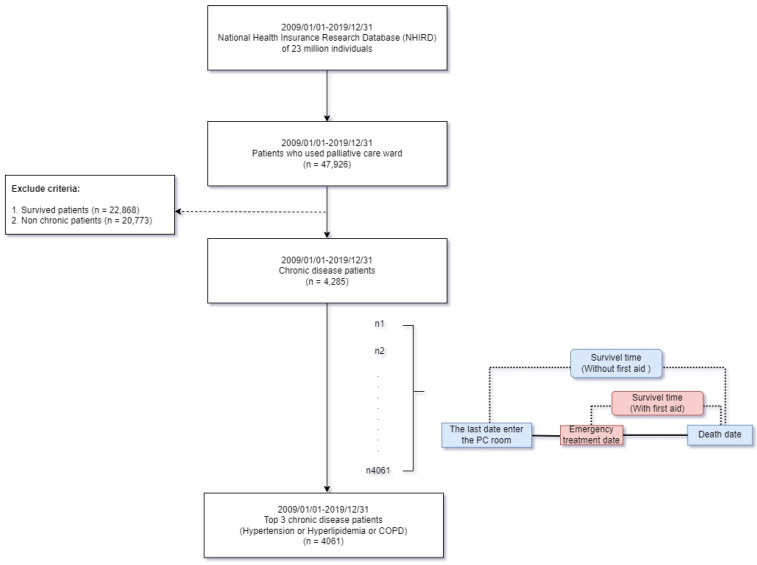
Selection flowchart.

As mentioned previously, the WHO defines chronic diseases as conditions that do not spread from person to person but persist for a long time and usually progress slowly; they are often irreversible and display symptoms that worsen over time and gradually limit an individual’s quality of life and ability to function [1]. This retrospective population-based cohort study included dead patients with chronic diseases who received PC services from 1 January 2009 to 31 December 2019, in Taiwan. Figure 1 presents the selection flowchart. This study selected patients who entered the PC ward between 1 January 2009 and 31 December 2019 (*n* = 47,926). Patients who had at least three outpatient clinic records and one inpatient record were enrolled in this study.

The exclusion criteria were as follows: survived patients (*n* = 22,868) and patients without chronic diseases (*n* = 20,773). Cancer and death records were extracted from TCR and the National Death Registry in Taiwan that is linked to the NHIRD database, and differences in the average total medical expenses of patients with chronic diseases from the time of the first utilization of PC services to death were examined [24,32].

In the next step, patients with chronic diseases who were admitted to PC wards were selected (*n* = 4285). Patients may have one or multiple chronic diseases, including hypertension (*n* = 2858), COPD (*n* = 2716), hyperlipidemia (*n* = 2588), diabetes (*n* = 2392), chronic kidney disease (CKD; *n* = 2005), chronic renal disease (CRD; *n* = 1710), liver cirrhosis (*n* = 572), and chronic pancreatitis (*n* = 69).

**Figure 2:**
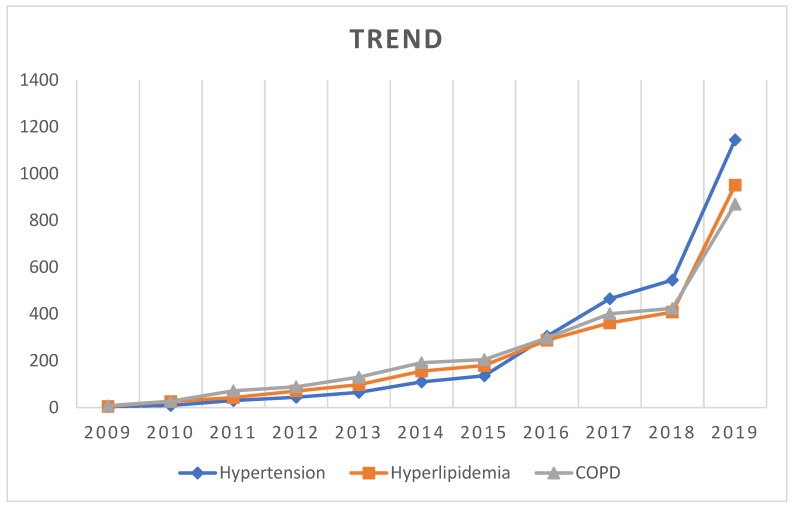
Trend in the number of patients with hypertension, hyperlipidemia, and COPD admitted to the PC wards during 2009–2019. COPD, chronic obstructive pulmonary disease.

Finally, the top three chronic diseases, namely hypertension, hyperlipidemia, and COPD, were selected as the target of this study and were detected in 4061 enrolled patients. The last PC ward admission date was considered as the index date, whereas a patient’s death was considered as the last follow-up time. Figure 2 indicates the trend in the number of patients with the three chronic diseases who were admitted to PC wards from 2009 to 2019. The highest number of patients reported COPD in 2011–2015, whereas the highest number of patients reported hypertension during 2016–2019.

### 2.3. Variable and Outcome Definitions

Admission to PC wards can be covered under health insurance in Taiwan; thus, there is a payment procedure code that enables identification in NHIRD. This research analyzed patients with chronic diseases who were admitted to PC wards. The mean survival time and total expenses of patients with chronic diseases from their last PC ward admission until death were the investigated outcomes. The total expenses included hospitalization, drug, emergency department, physician, examination, and PC service expenses. The following patient characteristics were investigated: sex (male/female), age (<65/65–79/≥80 years), and whether emergency treatment (cardiopulmonary resuscitation (CPR)/electric shock/intubation) was utilized. Moreover, the use of a urinary catheter, nasogastric tube, or respirator in their last PC ward admission was further investigated.

### 2.4. Statistical Analysis

This study selected patients with the top three chronic diseases who were admitted to the PC ward from 1 January 2009 to 31 December 2019, based on the demographic characteristics and medical information from the previous year. Chi-square test was used for all categorical data, which are expressed as numbers and percentages (*n* (%)). T-test was used for comparing continuous variables, which are presented as means and standard deviation (mean (SD)). Data were analyzed using SAS version 9.4 (SAS Institute Inc., Cary, NC, USA). All *p*-values were two-tailed; a value of <0.05 was considered statistically significant.

## 3. Results

In total, 4061 patients with the top three chronic diseases were admitted to the PC ward from 2009 to 2019, and 52.62% of these patients were men and 47.38% were women; furthermore, 44.00% of the patients were aged >85 years. The average age of the overall population was 78 (10.40) years. The proportion of patients with hypertension, hyperlipidemia, and COPD was 70.38%, 63.73%, and 66.88%, respectively, indicating that most patients had multiple chronic diseases. The average total medical expense for their last PC ward admission was NTD 199,173 (312,457) and the total hospitalization period was 9.63 (8.65) days, resulting in the total expense per day of NTD 38,936 (93,430) (Table 1).

Table 2 shows our two study groups. The first group comprised 3976 patients who did not receive emergency treatment after PC ward admission, whereas the second group included 85 patients who received emergency treatment. Among those who received emergency treatment, 51 (60%) were men and 34 (40%) were women. The proportion of patients aged <50, 50–64, 65–74, 75–84, and >85 years who selected emergency treatment was 7.06%, 11.76%, 20%, 9.41%, and 28.24%, respectively (*p* < 0.001 ***).

The average ages of the patients who did and did not receive emergency treatment were 72.83 (13.63) and 78.11 (10.30) years (*p* < 0.001 ***), respectively. The proportion of patients with hypertension who received emergency treatment was 81.70% (*p* < 0.001 ***). The average total expenses were NTD 190,945 (304,309) and NTD 584,088 (428,334) in patients without and with emergency treatment, respectively (*p* < 0.001 ***). The average hospitalization period of patients who received no emergency treatment was 9.64 (8.65) days, whereas that of patients who received emergency treatment was 9.05 (8.77) days.

Considering the emergency treatment history during the previous year before their final hospitalization, 8 patients underwent CPR, 3 received an electric shock, and 58 were intubated. Moreover, the life-sustaining treatments received by the patients during the final PC ward admission included urinary catheterization (600 (15.09%) patients without emergency treatment and 27 (31.76%) patients with emergency treatment; *p* < 0.001 ***), nasogastric intubation (747 (18.79%) and 56 (65.88%); *p* < 0.001 ***), and respirator use (148 (3.72%) and 74 (87.06), respectively; *p* < 0.001 ***).

In Table 3, among the patients admitted to the PC ward, male patients with emergency treatment showed higher average medical expense and shorter survival time than those without emergency treatment (NTD 580,387 (418,476), 97.39 (136.30) days vs. NTD 200,202 (309,943), 108.30 (234.40) days). Similarly, female patients with emergency treatment had higher average medical expense and shorter survival time than those without emergency treatment (NTD 589,640 (449,026), 69.56 (94.16) days vs. NTD 180,727 (297,715), 112.70 (260.80) days), as shown in Table 3.

Among different age groups, patients aged 50–64 years who received emergency treatment had higher emergency medical expense (NTD 753,754 (337,509) vs. NTD 247,602 (498,784)) and shorter survival time (183.9 (225.4) vs. 127.2 (343.9) days) than those who did not receive emergency treatment. Patients aged 65–74 years who received emergency treatment had higher emergency medical expense (NTD 608,391 (445,599) vs. NTD 189,850 (251,347)) and shorter survival time (73.4 (79.76) vs. 101.6 (248.6) days) than those who did not receive emergency treatment. Patients aged 75–84 years who received emergency treatment had higher emergency medical expense (NTD 480,429 (291,491) vs. NTD 197,827 (315,165)) and shorter survival time (70.76 (89.35) vs. 112.60 (248.10) days) than those who did not receive emergency treatment. Patients aged >85 years who received emergency treatment had higher emergency medical expense (NTD 444,092 (277,787) vs. NTD 164,795 (221,519)) and shorter survival time (53.58 (48.70) vs. 103.00 (207.90) days) than those who did not receive emergency treatment. In summary, patients aged <50 and >65 years survived longer if they did not receive emergency medical treatment, whereas those aged between 50 and 64 years showed the opposite result.

Table 4 shows that the top three chronic diseases were hypertension, hyperlipidemia, and COPD among the patients who developed multiple chronic diseases and were admitted to the PC ward. Each patient in this study might have multiple chronic diseases; 69 patients with hypertension received emergency treatment, whereas 2789 did not. The average age of patients with hypertension who received emergency treatment was 74.88 years. Overall, 25 of the 69 patients who received emergency treatment used a urinary catheter, 46 used a nasogastric tube, and 61 needed a respirator to maintain their lives. The average survival time of patients receiving emergency treatment was 81.26 days, and that of patients who did not receive it was longer at 105.60 days. Patients with emergency treatment had an average expense of NTD 560,141, which was higher than that of patients without emergency treatment (NTD 186,599).

The average age of 56 patients with hyperlipidemia receiving emergency treatment was 73.43 (13.53) years, whereas that of 2532 patients with hyperlipidemia not receiving emergency treatment was approximately 78.19 years. Of the 56 patients who received emergency treatment, 17 used a urinary catheter, 36 used a nasogastric tube, and 50 needed a respirator to maintain their lives. The average survival time of patients receiving emergency treatment was 86.82 days, and that of patients who did not receive emergency treatment was longer at 104.60 days. Patients with emergency treatment had an average expense of NTD 604,037, which was higher than that of patients without emergency treatment (NTD 179,743). A total of 54 patients with COPD received emergency treatment with an average age of 73.54 years, whereas 2662 did not. The average age of 2532 patients without emergency treatment was 79 years. Of the 54 patients who received emergency treatment, 11 used a urinary catheter, 32 used a nasogastric tube, and 46 needed a respirator to maintain their lives. The average survival time of patients receiving emergency treatment was 76.54 days, whereas that of patients who did not receive emergency treatment was longer at 122.20 days. Patients with emergency treatment had an average expense of NTD 555,774, which was higher than that of patients without emergency treatment (NTD 201,028).

## 4. Discussion

The main objective of PC is to help patients live as long as possible and to reduce physical and mental discomfort [33]. In addition, PC has been proven to enhance the patients’ quality of life. Thus, avoiding ineffective medical treatment at their end-of-life time is extremely important. Many researchers investigated the quality of life improvement and provide insight and guidance to patients with cancer at their end of life [34]; however, few studies have mentioned people with chronic diseases.

Chronic disease is a condition that lasts a year or more, requiring ongoing medical care or limiting activities of daily living [10]. Figure 2 shows an increasing trend of non-cancer chronic disease cases utilizing PC resources since 2009. Cancer treatments are covered under PC medical insurance since 1996. The eight major non-cancer chronic diseases added into the program were as follows: (1) elderly and presenile psychosis, (2) other brain deterioration, (3) heart failure, (4) COPD, (5) other respiratory diseases, (6) chronic liver disease and liver cirrhosis, (7) unspecified acute renal failure, and (8) unspecified chronic renal failure. This policy offered non-cancer patients an opportunity to obtain PC coverage by health insurance at the end of their lives. Figure 2 confirms that the health insurance policy establishment improved the usage of PC by terminal patients without cancer. Additionally, people who seek a good death are gradually gaining a consensus along with the popularized concept of the PC, leading to the rapid increase in PC need in patients without cancer. The present study revealed that 4061 patients have the top three chronic diseases, namely hypertension, hyperlipidemia, and COPD, and 85 patients received emergency treatment at the end of life.

As shown in Table 2, extremely few cases (85/4061 (2.09%)) still received emergency treatment, with a significance in age (*p* < 0.001). Despite being admitted to the PC ward, these patients and their family members decided to receive emergency treatments due to the difference between the age and average life expectancy in patients aged <50 years. Patients with emergency treatment had significantly higher inpatient medical expenses (NTD 143,223/day) than those without emergency treatment (NTD 36,663/day) (*p* < 0.001), whereas the average inpatient duration did not differ significantly.

PC can improve the quality of patient outcomes [24,35]. Furthermore, several studies have shown that PC reduced the average medical expense of patients with cancer and multiple comorbidities [28,36,37]. Our research shows that the average medical expense is lower in patients with emergency treatment than in those without any emergency treatment at the end of life. As shown in Table 4, the mean survival days in COPD and mean medical expense in each chronic disease were significantly different between patients without and with emergency treatment. Patients who received emergency treatment were more likely aged <75 years. The concept of PC is to offer a good death; however, the patients and their family members decided to receive emergency treatment because they consider that the patients are too young to die. In contrast, patients who did not receive emergency care were more likely aged approximately 80 years. For the PC ward patients who selected DNR, emergency treatment was not recommended regardless of their age. Emergency treatment requires a life support system, which is against the concept of PC.

PC ward patients receiving emergency treatment also received the following life support systems: urinary catheter (27/85 (31.76%)), nasogastric tube (56/85 (65.88%)), and respirator (74/85 = (87.06%)); however, a high percentage of PC patients did not receive emergency treatment (*p* < 0.001).

This indicates that patients still need a life support system to maintain their lives after receiving an emergency treatment, which is an obstacle for improving the quality of medical care at the end of life and offering a good death. Moreover, considering that there are cases with records of emergency treatment (CPR, electric shock, and tracheal intubation) who did not receive emergency treatment after PC ward admission, past experiences of suffering from the emergency treatment restrict the patients from selecting it later. Notably, some patients who were admitted to the hospice ward still accepted emergency treatment. Based on our study findings, it was found that, when the patient receives emergency treatment, their life is prolonged but the expense incurred is increased. In addition, the patients may have to endure the pain associated with the use of urinary catheter, nasogastric tube, and respirator. The benefit of PC is to reduce the physical, psychological, and spiritual suffering among patients [38]. Numerous studies have emphasized the concept of PC for acute diseases and cancer, but it is rarely indicated for chronic diseases. Physicians and nurses should aim to promote tranquility in patients with chronic diseases to relieve their pain.

## 5. Conclusions

The utilization of PC services was associated with fewer invasive procedures, lower hospitalization costs, and reduced medical staff burden. This study revealed that the promotion of tranquility in patients with chronic diseases is not comprehensive and that it is important to ensure optimal care and increased quality of life for patients with multiple chronic conditions.

## Figures and Tables

**Table 1:** Patients’ baseline characteristics.

Baseline	Overall Population (*n* = 4061)	
	N (%)	
Sex	Male	2137 (52.62%)
	Female	1924 (47.38%)
Age group (years)	<50	99 (2.44%)
	50–64	370 (9.11%)
	65–74	521 (12.83%)
	75–84	1284 (31.62%)
	≥85	1787 (44%)
Age (years)	78 (10.40)	
Hypertension	2858 (70.38%)	
Hyperlipidemia	2588 (63.73%)	
COPD	2716 (66.88%)	
Total medical expense	199,173 (312,457)	
LOS	9.63 (8.65)	
Total medical expense/LOS	38,936 (93,430)	

Abbreviations: COPD, chronic obstructive pulmonary disease; LOS, length of stay; CPR, cardiopulmonary resuscitation.

**Table 2:** Inpatients at end of life with and without emergency treatment.

Baseline		Without Emergency Treatment (*n* = 3976)	With Emergency Treatment (*n* = 85)	*p*
N (%)				
Sex	Male	2086 (52.46%)	51 (60%)	0.169
	Female	1890 (47.54%)	34 (40%)	
Age group (years)	<50	93 (2.34%)	6 (7.06%)	<0.001 ***
	50–64	360 (9.05%)	10 (11.76%)	
	65–74	501 (12.6%)	20 (23.53%)	
	75–84	1259 (31.66%)	25 (29.41%)	
	≥85	1763 (44.34%)	24 (28.24%)	
Age (years)		78.11 (10.30)	72.82 (13.63)	<0.001 ***
Hypertension		2789 (70.15%)	69 (81.70%)	0.028 *
Hyperlipidemia		2532 (63.68%)	56 (65.88%)	0.676
COPD		2662 (66.95%)	54 (63.53%)	0.507
Total medical expense		190,945 (304,309)	584,088 (428,334)	<0.001 ***
LOS		9.64 (8.65)	9.05 (8.77)	0.529
Total medical expense/LOS		36,663 (88,736)	143,223 (193,073)	<0.001 ***
**Previous 1 year**				
Emergency treatment 1 year before PC ward	CPR	8	-	-
	Electric shock	3	-	-
	Intubation	58	-	-
**Life support status**				
Vital signs	Urinary catheter	600 (15.09%)	27 (31.76%)	<0.001 ***
	Nasogastric tube	747 (18.79%)	56 (65.88%)	<0.001 ***
	Respirator	148 (3.72%)	74 (87.06%)	<0.001 ***

Abbreviations: COPD, chronic obstructive pulmonary disease; LOS, length of stay; CPR, cardiopulmonary resuscitation; *: *p* < 0.05; ***: *p* < 0.001.

**Table 3:** Survival time and medical expense.

Baseline		Overall			
		Total Medical Expense		Survival Time (Days)	
		Emergency Treatment		Emergency Treatment	
		No (*n* = 3976)	Yes (*n* = 85)	No (*n* = 3976)	Yes (*n* = 85)
Sex	Male	200,202 (309,943)	580,387 (418,476)	108.30 (234.40)	97.39 (136.30)
	Female	180,727 (297,715)	589,640 (449,026)	112.70 (260.80)	69.56 (94.16)
Age	<50	380,062 (571,354)	1,212,202 (823,866)	203.8 (404.1)	161.7 (223.2)
	50~64	247,602 (498,784)	753,754 (337,509)	127.2 (343.9)	183.9 (225.4)
	65–74	189,850 (251,347)	608,391 (445,599)	101.6 (248.6)	73.4 (79.76)
	75–84	197,827 (315,165)	480,429 (291,491)	112.60 (248.10)	70.76 (89.35)
	≥85	164,795 (221,519)	444,092 (277,787)	103.00 (207.90)	53.58 (48.70)

**Table 4:** Summary table of three chronic diseases.

Baseline		Top Three Chronic Diseases					
		Hypertension		Hyperlipidemia		COPD	
		Emergency Treatment		Emergency Treatment		Emergency Treatment	
		Yes (*n* = 2789)	No (*n* = 69)	Yes (*n* = 2532)	No (*n* = 56)	Yes (*n* = 2662)	No (*n* = 54)
Vital signs	Urinary catheter	397 (14.23%)	25 (36.23%)	385 (15.21%)	17 (30.36%)	356 (13.37%)	11 (20.37%)
	Nasogastric tube	490 (17.57%)	46 (66.67%)	460 (18.17%)	36 (64.29%)	464 (17.43%)	32 (59.26%)
	Respirator	77 (2.76%)	61 (88.41%)	92 (3.63%)	50 (89.29%)	103 (3.87%)	46 (85.19%)
Age (years)		79.30 (8.74)	74.88 (11.64)	78.19 (9.91)	73.43 (13.53)	79.00 (9.66)	73.54 (14.45)
Survival time (days)		105.60 (229.40)	81.26 (115.00)	104.60 (242.80)	86.82 (131.30)	122.20 (257.10)	76.54 (109.60)
Total medical expense		186,599 (310,259)	560,141 (374,140)	179,743 (262,900)	604,037 (421,797)	201,028 (305,875)	555,774 (437,261)

## Data Availability

Not applicable.
